# 4-[(4′-Chloro­methyl-[1,1′-biphen­yl]-4-yl)meth­yl]bis­(dimethyl­glyoximato-κ^2^ *N*,*N*′)(pyridine-κ*N*)cobalt(III)^1^

**DOI:** 10.1107/S1600536812001092

**Published:** 2012-01-14

**Authors:** Sarvendra Kumar, Suresh Thapa

**Affiliations:** a DQIAQF/INQUIMAE, Universidad de Buenos Aires, Ciudad Universitaria, Pab. II, p. 3, EHA1428 Buenos Aires, Argentina; b Faculty of Science and Technology, Purbanchal University, Biratnagar, Nepal

## Abstract

The title compound, [Co(C_14_H_14_Cl)(C_4_H_6_N_2_O_2_)_2_(C_5_H_5_N)], is a model compound for the more complex cobalamines like vitamins B_12_. The Co^III^ atom is coordinated by a (4′-chloro­methyl-[1,1′-biphen­yl]-4-yl)methyl group, an N-bonded pyridine and two *N*,*N*′-bidentate dimethyl­glyoximate ligands in a distorted octa­hedral geometry. The glyoximate ligands exhibit intra­molecular O—H⋯O hydrogen bonds, which is very common in cobaloxime derivatives.

## Related literature

For general background, see: Bresciani-Pahor *et al.* (1985 ▶); Revathi *et al.* (2009 ▶); Brown (2006 ▶); Randaccio (1999 ▶); For structure–property relationships, see: Gupta *et al.* (2004 ▶); Dutta *et al.* (2009 ▶). For a related structure, see: Kumar & Gupta (2011 ▶).
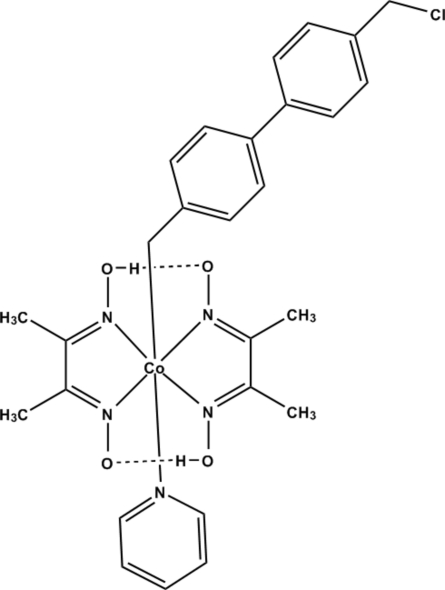


## Experimental

### 

#### Crystal data

[Co(C_14_H_14_Cl)(C_4_H_6_N_2_O_2_)_2_(C_5_H_5_N)]
*M* *_r_* = 583.95
Triclinic, 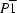

*a* = 9.1208 (15) Å
*b* = 11.3999 (19) Å
*c* = 13.661 (2) Å
α = 72.869 (3)°
β = 77.504 (3)°
γ = 87.276 (3)°
*V* = 1325.1 (4) Å^3^
*Z* = 2
Mo *K*α radiation
μ = 0.79 mm^−1^
*T* = 100 K
0.32 × 0.28 × 0.26 mm

#### Data collection

Bruker SMART CCD area-detector diffractometer
Absorption correction: multi-scan (*SADABS*; Sheldrick, 2004 ▶) *T*_min_ = 0.786, *T*_max_ = 0.821
7047 measured reflections
4789 independent reflections
3996 reflections with *I* > 2σ(*I*)
*R*_int_ = 0.022

#### Refinement

*R*[*F* ^2^ > 2σ(*F* ^2^)] = 0.047
*wR*(*F* ^2^) = 0.125
*S* = 1.04
4789 reflections
349 parameters
H-atom parameters constrained
Δρ_max_ = 0.65 e Å^−3^
Δρ_min_ = −0.43 e Å^−3^

### 

Data collection: *SMART* (Bruker, 2001 ▶); cell refinement: *SAINT* (Bruker, 2001 ▶); data reduction: *SAINT*; program(s) used to solve structure: *SHELXS97* (Sheldrick, 2008 ▶); program(s) used to refine structure: *SHELXL97* (Sheldrick, 2008 ▶); molecular graphics: *SHELXTL* (Sheldrick, 2008 ▶); software used to prepare material for publication: *DIAMOND* (Brandenburg, 1999 ▶).

## Supplementary Material

Crystal structure: contains datablock(s) I, global. DOI: 10.1107/S1600536812001092/fk2049sup1.cif

Structure factors: contains datablock(s) I. DOI: 10.1107/S1600536812001092/fk2049Isup2.hkl

Additional supplementary materials:  crystallographic information; 3D view; checkCIF report

## Figures and Tables

**Table 1:** Selected bond lengths (Å)

Co1—N1	1.875 (2)
Co1—N2	1.877 (2)
Co1—N3	1.879 (2)
Co1—N4	1.875 (2)
Co1—N5	2.055 (2)

**Table 2:** Hydrogen-bond geometry (Å, °)

*D*—H⋯*A*	*D*—H	H⋯*A*	*D*⋯*A*	*D*—H⋯*A*
O2—H2⋯O4	0.84	1.67	2.479 (3)	161
O3—H3⋯O1	0.84	1.67	2.478 (3)	160

**Fig. 1.**
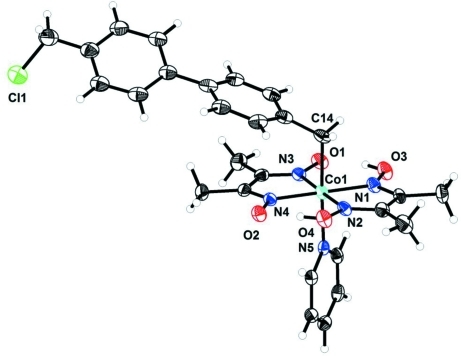
Molecular structure of the title compound with displacement ellipsoids drawn at the 50% probability level for non-hydrogen atoms.

**Fig. 2.**
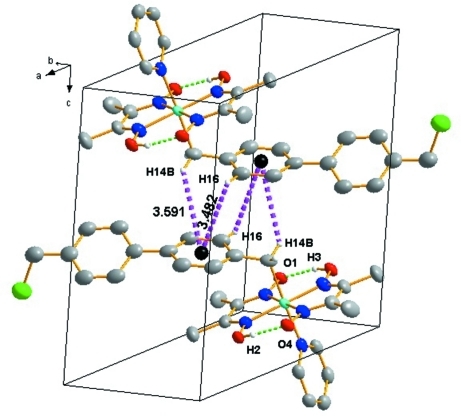
Crystal packing. Dotted lines represent intramolecular O—H···O and intermolecular C—H···π interactions.

## supplementary crystallographic information

### Comment

The chemistry and molecular structure of bis(dimethylglyoximato)cobalt(III) complexes, trivially known as cobaloximes (Bresciani-Pahor *et al.*, 1985), have been of great interest to chemists for the past four decades for two reasons. First, the coordination chemistry of these complexes is far-reaching, with almost unlimited possibilities for substituents in the axial position and variation in the equatorial ligands (Brown, 2006; Randaccio 1999). Second, many organometallic cobaloxime derivatives have been used as model compounds for the study of vitamin B_12_ coenzyme. Cobaloximes have played its role in helping to understand the reactivity of the cobalt-carbon bond (Gupta *et al.*,2004; Dutta *et al.*, 2009). The inherently weak Co—C bond in the organocobaloximes undergoes homolytic cleavage with visible light, similar to the activation of vitamin B_12_ by apoenzyme and have been utilized in organic synthesis, catalysis and in polymer chemistry. Most of the recent studies on cobaloximes have been focused on their structure-property relationships (Gupta *et al.*, 2004). Herein, we have reported the synthesis and structure of a new cobaloxime.

The crystal structure of the title compound is shown in Figure 1. The coordination of cobalt(III) ion is slightly distorted octahedral (Revathi *et al.*, 2009) with the aryl group, the 4-((4'-(chloromethyl)-[1,1'-biphenyl]-4-yl)methyl group, a pyridine ligand and two N,*N*-bidentate dimethylglyoximate ligands. The Co—N(dmg) bond lengths range from 1.873 (2) to 1.880 (2) Å. The bite angles N3—Co1—N4 and N1—Co1—N2 of the ligand are 81.45 (11)° and 81.44 (11)°, respectively. The coordinated 4-((4'-(chloromethyl)-[1,1'-biphenyl]-4-yl)methyl group and the pyridine ring nitrogen are coordinated axially in trans position with the angle C14—Co1—N5 = 177.88 (9)°. The important bond lengths and bond angles are given in Table 1, and intramolecular hydrogen bonding parameters are given in Table 2. The two glyoximate moieties are linked together by strong intramolecular O–H···O hydrogen bonding (Fig. 2). Additionally, the packing (Fig. 2) shows molecules bonded through C-H···π interactions within 3.4824 (4) - 3.5907 (5) Å.

### Experimental

A solution of ClCo(dmgH)_2_py (1 mmol) in 10 ml of methanol was purged thoroughly with N_2_ for 20 min and was cooled to 0°C with stirring. The solution turned deep blue after the addition of a few drops of aqueous NaOH followed by sodium borohydride (1.5 mmol in 0.5 ml of water). The colour of the solution turned orange-red on addition of 4,4'-bis(chloromethyl)-1,1'-biphenyl (1 mmol). The reaction was stirred 1 h at 0°C then poured into 20 ml chilled water. The resulting orange-red precipitate was filtered, washed with water, and dried. The obtained orange coloured compound was recrystallized from dichloromethane and methanol. After five days, orange coloured crystals were obtained, suitable for single-crystal data collection.

### Refinement

All H atoms were derived from difference Fourier maps and then refined at idealized positions riding with C—H 0.95 – 0.99 Å, O–H 0.84 Å and *U*_iso_ = 1.2 *U*_eq_(C) or 1.5 (C-methyl and O).

### Crystal data

**Table:**

[Co(C_14_H_14_Cl)(C_4_H_6_N_2_O_2_)_2_(C_5_H_5_N)]	*Z* = 2
*M**_r_* = 583.95	*F*(000) = 608
Triclinic, *P*1	*D*_x_ = 1.464 Mg m^−^^3^
Hall symbol: -P 1	Mo *K*α radiation, λ = 0.71073 Å
*a* = 9.1208 (15) Å	Cell parameters from 2484 reflections
*b* = 11.3999 (19) Å	θ = 2.8–27.6°
*c* = 13.661 (2) Å	µ = 0.79 mm^−^^1^
α = 72.869 (3)°	*T* = 100 K
β = 77.504 (3)°	Prism, orange
γ = 87.276 (3)°	0.32 × 0.28 × 0.26 mm
*V* = 1325.1 (4) Å^3^	

### Data collection

**Table:**

Bruker SMART CCD area-detector diffractometer	4789 independent reflections
Radiation source: fine-focus sealed tube	3996 reflections with *I* > 2σ(*I*)
graphite	*R*_int_ = 0.022
phi and ω scans	θ_max_ = 25.5°, θ_min_ = 2.1°
Absorption correction: multi-scan (*SADABS*; Sheldrick, 2004)	*h* = −11→11
*T*_min_ = 0.786, *T*_max_ = 0.821	*k* = −13→13
7047 measured reflections	*l* = −9→16

### Refinement

**Table:**

Refinement on *F*^2^	Primary atom site location: structure-invariant direct methods
Least-squares matrix: full	Secondary atom site location: difference Fourier map
*R*[*F*^2^ > 2σ(*F*^2^)] = 0.047	Hydrogen site location: inferred from neighbouring sites
*wR*(*F*^2^) = 0.125	H-atom parameters constrained
*S* = 1.04	*w* = 1/[σ^2^(*F*_o_^2^) + (0.0624*P*)^2^ + 0.7675*P*] where *P* = (*F*_o_^2^ + 2*F*_c_^2^)/3
4789 reflections	(Δ/σ)_max_ = 0.014
349 parameters	Δρ_max_ = 0.65 e Å^−^^3^
0 restraints	Δρ_min_ = −0.43 e Å^−^^3^

### Special details

**Table:**

Geometry. All s.u.'s (except the s.u. in the dihedral angle between two l.s. planes) are estimated using the full covariance matrix. The cell s.u.'s are taken into account individually in the estimation of s.u.'s in distances, angles and torsion angles; correlations between s.u.'s in cell parameters are only used when they are defined by crystal symmetry. An approximate (isotropic) treatment of cell s.u.'s is used for estimating s.u.'s involving l.s. planes.
Refinement. Refinement of *F*^2^ against ALL reflections. The weighted *R*-factor *wR* and goodness of fit *S* are based on *F*^2^, conventional *R*-factors *R* are based on *F*, with *F* set to zero for negative *F*^2^. The threshold expression of *F*^2^ > 2σ(*F*^2^) is used only for calculating *R*-factors(gt) *etc*. and is not relevant to the choice of reflections for refinement. *R*-factors based on *F*^2^ are statistically about twice as large as those based on *F*, and *R*- factors based on ALL data will be even larger.

### Fractional atomic coordinates and isotropic or equivalent isotropic displacement parameters (Å^2^)

**Table:**

	*x*	*y*	*z*	*U*_iso_*/*U*_eq_	
C1	0.5467 (3)	0.4296 (3)	0.8689 (2)	0.0244 (6)	
C2	0.4200 (3)	0.5114 (2)	0.8484 (2)	0.0236 (6)	
C3	0.6928 (3)	0.4741 (3)	0.8776 (2)	0.0328 (7)	
H3A	0.7613	0.4051	0.8907	0.049*	
H3B	0.6763	0.5104	0.9358	0.049*	
H3C	0.7369	0.5362	0.8122	0.049*	
C4	0.4242 (4)	0.6451 (3)	0.8370 (3)	0.0353 (7)	
H4A	0.3245	0.6793	0.8322	0.053*	
H4B	0.4972	0.6864	0.7733	0.053*	
H4C	0.4537	0.6578	0.8980	0.053*	
C5	0.2347 (3)	0.0577 (3)	0.8636 (2)	0.0267 (6)	
C6	0.1069 (3)	0.1387 (3)	0.8459 (2)	0.0259 (6)	
C7	0.2347 (4)	−0.0776 (3)	0.8776 (3)	0.0405 (8)	
H7A	0.3159	−0.0966	0.8249	0.061*	
H7B	0.1381	−0.1030	0.8691	0.061*	
H7C	0.2500	−0.1216	0.9478	0.061*	
C8	−0.0405 (4)	0.0952 (3)	0.8371 (3)	0.0361 (7)	
H8A	−0.1123	0.1623	0.8330	0.054*	
H8B	−0.0790	0.0261	0.8987	0.054*	
H8C	−0.0268	0.0687	0.7737	0.054*	
C9	0.1205 (3)	0.2947 (2)	1.0542 (2)	0.0236 (6)	
H9	0.0680	0.3500	1.0074	0.028*	
C10	0.0644 (3)	0.2700 (2)	1.1611 (2)	0.0252 (6)	
H10	−0.0247	0.3078	1.1869	0.030*	
C11	0.1406 (3)	0.1892 (2)	1.2298 (2)	0.0261 (6)	
H11	0.1055	0.1713	1.3035	0.031*	
C12	0.2681 (3)	0.1354 (2)	1.1890 (2)	0.0249 (6)	
H12	0.3219	0.0790	1.2342	0.030*	
C13	0.3172 (3)	0.1641 (2)	1.0815 (2)	0.0216 (6)	
H13	0.4051	0.1260	1.0542	0.026*	
C14	0.4029 (3)	0.3241 (3)	0.6945 (2)	0.0280 (6)	
H14A	0.4287	0.2461	0.6773	0.034*	
H14B	0.3195	0.3607	0.6603	0.034*	
C15	0.5346 (4)	0.4084 (3)	0.6493 (2)	0.0300 (7)	
C16	0.5165 (3)	0.5349 (3)	0.6105 (2)	0.0301 (7)	
H16	0.4186	0.5656	0.6066	0.036*	
C17	0.6360 (3)	0.6165 (3)	0.5778 (2)	0.0311 (7)	
H17	0.6188	0.7019	0.5526	0.037*	
C18	0.7833 (3)	0.5753 (3)	0.5810 (2)	0.0297 (7)	
C19	0.8023 (4)	0.4479 (3)	0.6162 (2)	0.0322 (7)	
H19	0.9004	0.4166	0.6178	0.039*	
C20	0.6826 (4)	0.3678 (3)	0.6480 (2)	0.0313 (7)	
H20	0.7000	0.2821	0.6700	0.038*	
C21	0.9099 (3)	0.6632 (3)	0.5546 (2)	0.0299 (7)	
C22	0.9150 (4)	0.7773 (3)	0.4799 (2)	0.0335 (7)	
H22	0.8370	0.7989	0.4420	0.040*	
C23	1.0328 (4)	0.8605 (3)	0.4598 (2)	0.0372 (8)	
H23	1.0331	0.9384	0.4094	0.045*	
C24	1.1498 (4)	0.8307 (3)	0.5128 (2)	0.0361 (7)	
C25	1.1457 (3)	0.7166 (3)	0.5874 (2)	0.0346 (7)	
H25	1.2244	0.6946	0.6246	0.041*	
C26	1.0279 (3)	0.6349 (3)	0.6076 (2)	0.0331 (7)	
H26	1.0271	0.5575	0.6589	0.040*	
C27	1.2793 (4)	0.9190 (4)	0.4875 (3)	0.0467 (9)	
H27A	1.2576	0.9971	0.4376	0.056*	
H27B	1.3707	0.8846	0.4528	0.056*	
N1	0.1383 (3)	0.2514 (2)	0.83606 (18)	0.0246 (5)	
N2	0.3515 (3)	0.1170 (2)	0.86675 (18)	0.0229 (5)	
N3	0.3069 (3)	0.4537 (2)	0.83950 (17)	0.0223 (5)	
N4	0.5175 (2)	0.3177 (2)	0.87419 (17)	0.0216 (5)	
N5	0.2458 (2)	0.24374 (19)	1.01403 (18)	0.0204 (5)	
O1	0.1808 (2)	0.51432 (17)	0.81945 (16)	0.0291 (5)	
O2	0.6233 (2)	0.23031 (18)	0.89279 (16)	0.0277 (5)	
H2	0.5929	0.1641	0.8882	0.042*	
O3	0.0329 (2)	0.33869 (18)	0.81727 (17)	0.0313 (5)	
H3	0.0696	0.4077	0.8099	0.047*	
O4	0.4805 (2)	0.05861 (17)	0.87842 (16)	0.0289 (5)	
Cl1	1.31444 (11)	0.94962 (9)	0.60281 (7)	0.0528 (3)	
Co1	0.32783 (4)	0.28473 (3)	0.85516 (3)	0.02023 (14)	

### Atomic displacement parameters (Å^2^)

**Table:**

	*U*^11^	*U*^22^	*U*^33^	*U*^12^	*U*^13^	*U*^23^
C1	0.0278 (15)	0.0268 (15)	0.0186 (13)	−0.0066 (12)	−0.0018 (11)	−0.0077 (11)
C2	0.0338 (16)	0.0185 (14)	0.0185 (13)	−0.0031 (12)	−0.0044 (12)	−0.0056 (11)
C3	0.0286 (16)	0.0409 (19)	0.0311 (16)	−0.0104 (14)	−0.0057 (13)	−0.0129 (14)
C4	0.050 (2)	0.0193 (15)	0.0385 (18)	−0.0059 (14)	−0.0092 (15)	−0.0105 (13)
C5	0.0354 (17)	0.0206 (14)	0.0276 (15)	−0.0019 (12)	−0.0099 (13)	−0.0099 (12)
C6	0.0293 (15)	0.0261 (15)	0.0269 (15)	−0.0042 (12)	−0.0109 (12)	−0.0104 (12)
C7	0.061 (2)	0.0227 (16)	0.045 (2)	−0.0017 (15)	−0.0239 (17)	−0.0126 (14)
C8	0.0325 (17)	0.0388 (18)	0.0441 (19)	−0.0069 (14)	−0.0158 (15)	−0.0160 (15)
C9	0.0239 (14)	0.0170 (13)	0.0333 (16)	0.0013 (11)	−0.0123 (12)	−0.0082 (12)
C10	0.0214 (14)	0.0198 (14)	0.0369 (16)	0.0003 (11)	−0.0059 (12)	−0.0122 (12)
C11	0.0282 (15)	0.0213 (14)	0.0283 (15)	−0.0026 (12)	−0.0049 (12)	−0.0066 (12)
C12	0.0292 (15)	0.0180 (14)	0.0286 (15)	−0.0005 (11)	−0.0118 (12)	−0.0041 (11)
C13	0.0201 (14)	0.0161 (13)	0.0301 (15)	0.0026 (10)	−0.0079 (11)	−0.0074 (11)
C14	0.0381 (17)	0.0275 (15)	0.0229 (14)	0.0062 (13)	−0.0121 (13)	−0.0110 (12)
C15	0.0409 (18)	0.0292 (16)	0.0218 (14)	0.0044 (13)	−0.0090 (13)	−0.0094 (12)
C16	0.0333 (17)	0.0332 (17)	0.0239 (15)	0.0115 (13)	−0.0091 (13)	−0.0082 (13)
C17	0.0385 (18)	0.0296 (16)	0.0238 (15)	0.0063 (13)	−0.0092 (13)	−0.0046 (13)
C18	0.0379 (17)	0.0299 (16)	0.0197 (14)	0.0083 (13)	−0.0060 (12)	−0.0061 (12)
C19	0.0357 (17)	0.0350 (17)	0.0246 (15)	0.0092 (14)	−0.0042 (13)	−0.0097 (13)
C20	0.0412 (18)	0.0260 (16)	0.0251 (15)	0.0071 (13)	−0.0039 (13)	−0.0083 (12)
C21	0.0308 (16)	0.0323 (16)	0.0246 (15)	0.0082 (13)	−0.0032 (12)	−0.0087 (13)
C22	0.0360 (17)	0.0364 (18)	0.0255 (15)	0.0075 (14)	−0.0072 (13)	−0.0059 (13)
C23	0.0402 (19)	0.0395 (19)	0.0266 (16)	0.0009 (15)	−0.0056 (14)	−0.0029 (14)
C24	0.0327 (17)	0.046 (2)	0.0271 (16)	0.0041 (14)	−0.0009 (13)	−0.0116 (14)
C25	0.0304 (17)	0.0438 (19)	0.0275 (16)	0.0110 (14)	−0.0037 (13)	−0.0107 (14)
C26	0.0346 (17)	0.0366 (18)	0.0248 (15)	0.0117 (14)	−0.0027 (13)	−0.0080 (13)
C27	0.042 (2)	0.060 (2)	0.0338 (18)	−0.0021 (17)	−0.0076 (15)	−0.0055 (17)
N1	0.0252 (12)	0.0218 (12)	0.0305 (13)	0.0056 (10)	−0.0133 (10)	−0.0086 (10)
N2	0.0261 (12)	0.0210 (12)	0.0255 (12)	0.0051 (10)	−0.0117 (10)	−0.0091 (10)
N3	0.0279 (12)	0.0188 (12)	0.0216 (12)	0.0014 (9)	−0.0089 (10)	−0.0057 (9)
N4	0.0217 (12)	0.0220 (12)	0.0214 (12)	0.0012 (9)	−0.0070 (9)	−0.0054 (9)
N5	0.0186 (11)	0.0154 (11)	0.0287 (12)	0.0000 (9)	−0.0082 (9)	−0.0062 (9)
O1	0.0333 (11)	0.0208 (10)	0.0357 (11)	0.0122 (8)	−0.0167 (9)	−0.0071 (9)
O2	0.0221 (10)	0.0277 (11)	0.0356 (11)	0.0059 (8)	−0.0124 (9)	−0.0091 (9)
O3	0.0299 (11)	0.0264 (11)	0.0437 (13)	0.0090 (9)	−0.0201 (10)	−0.0120 (10)
O4	0.0310 (11)	0.0223 (10)	0.0386 (12)	0.0131 (8)	−0.0161 (9)	−0.0121 (9)
Cl1	0.0548 (6)	0.0574 (6)	0.0447 (5)	−0.0059 (5)	−0.0189 (4)	−0.0054 (4)
Co1	0.0228 (2)	0.0156 (2)	0.0257 (2)	0.00326 (15)	−0.01128 (16)	−0.00724 (15)

### Geometric parameters (Å, °)

**Table:**

C1—N4	1.294 (4)	C15—C16	1.398 (4)
C1—C2	1.472 (4)	C15—C20	1.404 (4)
C1—C3	1.489 (4)	C16—C17	1.376 (4)
C2—N3	1.295 (4)	C16—H16	0.9500
C2—C4	1.487 (4)	C17—C18	1.408 (4)
C3—H3A	0.9800	C17—H17	0.9500
C3—H3B	0.9800	C18—C19	1.404 (4)
C3—H3C	0.9800	C18—C21	1.477 (4)
C4—H4A	0.9800	C19—C20	1.367 (4)
C4—H4B	0.9800	C19—H19	0.9500
C4—H4C	0.9800	C20—H20	0.9500
C5—N2	1.304 (4)	C21—C22	1.393 (4)
C5—C6	1.469 (4)	C21—C26	1.398 (4)
C5—C7	1.497 (4)	C22—C23	1.395 (5)
C6—N1	1.291 (4)	C22—H22	0.9500
C6—C8	1.495 (4)	C23—C24	1.390 (4)
C7—H7A	0.9800	C23—H23	0.9500
C7—H7B	0.9800	C24—C25	1.392 (4)
C7—H7C	0.9800	C24—C27	1.503 (5)
C8—H8A	0.9800	C25—C26	1.383 (5)
C8—H8B	0.9800	C25—H25	0.9500
C8—H8C	0.9800	C26—H26	0.9500
C9—N5	1.341 (4)	C27—Cl1	1.805 (4)
C9—C10	1.387 (4)	C27—H27A	0.9900
C9—H9	0.9500	C27—H27B	0.9900
C10—C11	1.387 (4)	N1—O3	1.359 (3)
C10—H10	0.9500	N1—Co1	1.875 (2)
C11—C12	1.376 (4)	N2—O4	1.340 (3)
C11—H11	0.9500	N2—Co1	1.877 (2)
C12—C13	1.382 (4)	N3—O1	1.351 (3)
C12—H12	0.9500	N3—Co1	1.879 (2)
C13—N5	1.345 (3)	N4—O2	1.362 (3)
C13—H13	0.9500	N4—Co1	1.875 (2)
C14—C15	1.479 (4)	N5—Co1	2.055 (2)
C14—Co1	2.071 (3)	O2—H2	0.8400
C14—H14A	0.9900	O3—H3	0.8400
C14—H14B	0.9900		
			
N4—C1—C2	112.3 (2)	C19—C18—C17	116.9 (3)
N4—C1—C3	124.9 (3)	C19—C18—C21	122.0 (3)
C2—C1—C3	122.8 (3)	C17—C18—C21	121.0 (3)
N3—C2—C1	112.0 (2)	C20—C19—C18	121.5 (3)
N3—C2—C4	124.5 (3)	C20—C19—H19	119.3
C1—C2—C4	123.5 (3)	C18—C19—H19	119.3
C1—C3—H3A	109.5	C19—C20—C15	122.0 (3)
C1—C3—H3B	109.5	C19—C20—H20	119.0
H3A—C3—H3B	109.5	C15—C20—H20	119.0
C1—C3—H3C	109.5	C22—C21—C26	117.5 (3)
H3A—C3—H3C	109.5	C22—C21—C18	122.4 (3)
H3B—C3—H3C	109.5	C26—C21—C18	120.1 (3)
C2—C4—H4A	109.5	C21—C22—C23	121.1 (3)
C2—C4—H4B	109.5	C21—C22—H22	119.5
H4A—C4—H4B	109.5	C23—C22—H22	119.5
C2—C4—H4C	109.5	C24—C23—C22	120.7 (3)
H4A—C4—H4C	109.5	C24—C23—H23	119.6
H4B—C4—H4C	109.5	C22—C23—H23	119.6
N2—C5—C6	112.2 (2)	C23—C24—C25	118.5 (3)
N2—C5—C7	122.5 (3)	C23—C24—C27	120.3 (3)
C6—C5—C7	125.3 (3)	C25—C24—C27	121.2 (3)
N1—C6—C5	112.2 (2)	C26—C25—C24	120.5 (3)
N1—C6—C8	124.3 (3)	C26—C25—H25	119.8
C5—C6—C8	123.5 (3)	C24—C25—H25	119.8
C5—C7—H7A	109.5	C25—C26—C21	121.7 (3)
C5—C7—H7B	109.5	C25—C26—H26	119.2
H7A—C7—H7B	109.5	C21—C26—H26	119.2
C5—C7—H7C	109.5	C24—C27—Cl1	112.3 (2)
H7A—C7—H7C	109.5	C24—C27—H27A	109.1
H7B—C7—H7C	109.5	Cl1—C27—H27A	109.1
C6—C8—H8A	109.5	C24—C27—H27B	109.1
C6—C8—H8B	109.5	Cl1—C27—H27B	109.1
H8A—C8—H8B	109.5	H27A—C27—H27B	107.9
C6—C8—H8C	109.5	C6—N1—O3	119.7 (2)
H8A—C8—H8C	109.5	C6—N1—Co1	117.25 (19)
H8B—C8—H8C	109.5	O3—N1—Co1	123.03 (17)
N5—C9—C10	122.7 (2)	C5—N2—O4	120.5 (2)
N5—C9—H9	118.6	C5—N2—Co1	116.67 (19)
C10—C9—H9	118.6	O4—N2—Co1	122.87 (17)
C11—C10—C9	118.9 (3)	C2—N3—O1	120.4 (2)
C11—C10—H10	120.5	C2—N3—Co1	117.09 (19)
C9—C10—H10	120.5	O1—N3—Co1	122.48 (17)
C12—C11—C10	118.6 (3)	C1—N4—O2	119.6 (2)
C12—C11—H11	120.7	C1—N4—Co1	117.2 (2)
C10—C11—H11	120.7	O2—N4—Co1	123.19 (17)
C11—C12—C13	119.4 (3)	C9—N5—C13	117.7 (2)
C11—C12—H12	120.3	C9—N5—Co1	121.72 (18)
C13—C12—H12	120.3	C13—N5—Co1	120.57 (19)
N5—C13—C12	122.7 (3)	N4—O2—H2	109.5
N5—C13—H13	118.6	N1—O3—H3	109.5
C12—C13—H13	118.6	N4—Co1—N1	179.86 (10)
C15—C14—Co1	115.2 (2)	N4—Co1—N2	98.40 (10)
C15—C14—H14A	108.5	N1—Co1—N2	81.46 (10)
Co1—C14—H14A	108.5	N4—Co1—N3	81.41 (10)
C15—C14—H14B	108.5	N1—Co1—N3	98.72 (10)
Co1—C14—H14B	108.5	N2—Co1—N3	178.36 (10)
H14A—C14—H14B	107.5	N4—Co1—N5	89.74 (9)
C16—C15—C20	116.5 (3)	N1—Co1—N5	90.30 (10)
C16—C15—C14	120.9 (3)	N2—Co1—N5	90.44 (9)
C20—C15—C14	122.6 (3)	N3—Co1—N5	91.19 (9)
C17—C16—C15	122.1 (3)	N4—Co1—C14	92.37 (11)
C17—C16—H16	119.0	N1—Co1—C14	87.59 (11)
C15—C16—H16	119.0	N2—Co1—C14	89.08 (11)
C16—C17—C18	121.0 (3)	N3—Co1—C14	89.30 (11)
C16—C17—H17	119.5	N5—Co1—C14	177.89 (10)
C18—C17—H17	119.5		
			
N4—C1—C2—N3	−0.4 (3)	C12—C13—N5—C9	−1.2 (4)
C3—C1—C2—N3	177.2 (2)	C12—C13—N5—Co1	178.7 (2)
N4—C1—C2—C4	−179.1 (2)	C1—N4—Co1—N1	162 (100)
C3—C1—C2—C4	−1.4 (4)	O2—N4—Co1—N1	−17 (50)
N2—C5—C6—N1	0.8 (4)	C1—N4—Co1—N2	178.4 (2)
C7—C5—C6—N1	−179.7 (3)	O2—N4—Co1—N2	−1.4 (2)
N2—C5—C6—C8	178.9 (3)	C1—N4—Co1—N3	0.1 (2)
C7—C5—C6—C8	−1.5 (5)	O2—N4—Co1—N3	−179.8 (2)
N5—C9—C10—C11	−0.1 (4)	C1—N4—Co1—N5	−91.2 (2)
C9—C10—C11—C12	−0.8 (4)	O2—N4—Co1—N5	89.0 (2)
C10—C11—C12—C13	0.7 (4)	C1—N4—Co1—C14	89.0 (2)
C11—C12—C13—N5	0.4 (4)	O2—N4—Co1—C14	−90.9 (2)
Co1—C14—C15—C16	−92.7 (3)	C6—N1—Co1—N4	21 (50)
Co1—C14—C15—C20	83.7 (3)	O3—N1—Co1—N4	−162 (100)
C20—C15—C16—C17	−3.3 (4)	C6—N1—Co1—N2	4.5 (2)
C14—C15—C16—C17	173.3 (3)	O3—N1—Co1—N2	−178.4 (2)
C15—C16—C17—C18	0.7 (5)	C6—N1—Co1—N3	−177.1 (2)
C16—C17—C18—C19	1.7 (4)	O3—N1—Co1—N3	−0.1 (2)
C16—C17—C18—C21	−174.7 (3)	C6—N1—Co1—N5	−85.9 (2)
C17—C18—C19—C20	−1.5 (4)	O3—N1—Co1—N5	91.2 (2)
C21—C18—C19—C20	174.9 (3)	C6—N1—Co1—C14	93.9 (2)
C18—C19—C20—C15	−1.2 (5)	O3—N1—Co1—C14	−89.0 (2)
C16—C15—C20—C19	3.5 (4)	C5—N2—Co1—N4	176.0 (2)
C14—C15—C20—C19	−173.0 (3)	O4—N2—Co1—N4	−3.5 (2)
C19—C18—C21—C22	150.8 (3)	C5—N2—Co1—N1	−4.0 (2)
C17—C18—C21—C22	−33.0 (4)	O4—N2—Co1—N1	176.4 (2)
C19—C18—C21—C26	−31.4 (4)	C5—N2—Co1—N3	−101 (3)
C17—C18—C21—C26	144.9 (3)	O4—N2—Co1—N3	80 (3)
C26—C21—C22—C23	−0.7 (5)	C5—N2—Co1—N5	86.2 (2)
C18—C21—C22—C23	177.2 (3)	O4—N2—Co1—N5	−93.3 (2)
C21—C22—C23—C24	1.1 (5)	C5—N2—Co1—C14	−91.7 (2)
C22—C23—C24—C25	−0.8 (5)	O4—N2—Co1—C14	88.7 (2)
C22—C23—C24—C27	177.6 (3)	C2—N3—Co1—N4	−0.3 (2)
C23—C24—C25—C26	0.2 (5)	O1—N3—Co1—N4	179.4 (2)
C27—C24—C25—C26	−178.1 (3)	C2—N3—Co1—N1	179.7 (2)
C24—C25—C26—C21	0.1 (5)	O1—N3—Co1—N1	−0.5 (2)
C22—C21—C26—C25	0.1 (4)	C2—N3—Co1—N2	−84 (3)
C18—C21—C26—C25	−177.9 (3)	O1—N3—Co1—N2	96 (3)
C23—C24—C27—Cl1	127.7 (3)	C2—N3—Co1—N5	89.2 (2)
C25—C24—C27—Cl1	−53.9 (4)	O1—N3—Co1—N5	−91.0 (2)
C5—C6—N1—O3	178.8 (2)	C2—N3—Co1—C14	−92.8 (2)
C8—C6—N1—O3	0.6 (4)	O1—N3—Co1—C14	86.9 (2)
C5—C6—N1—Co1	−4.1 (3)	C9—N5—Co1—N4	129.1 (2)
C8—C6—N1—Co1	177.8 (2)	C13—N5—Co1—N4	−50.9 (2)
C6—C5—N2—O4	−177.5 (2)	C9—N5—Co1—N1	−51.0 (2)
C7—C5—N2—O4	2.9 (4)	C13—N5—Co1—N1	129.0 (2)
C6—C5—N2—Co1	2.9 (3)	C9—N5—Co1—N2	−132.5 (2)
C7—C5—N2—Co1	−176.7 (2)	C13—N5—Co1—N2	47.5 (2)
C1—C2—N3—O1	−179.3 (2)	C9—N5—Co1—N3	47.7 (2)
C4—C2—N3—O1	−0.6 (4)	C13—N5—Co1—N3	−132.3 (2)
C1—C2—N3—Co1	0.5 (3)	C9—N5—Co1—C14	−56 (3)
C4—C2—N3—Co1	179.1 (2)	C13—N5—Co1—C14	124 (3)
C2—C1—N4—O2	−180.0 (2)	C15—C14—Co1—N4	−25.2 (2)
C3—C1—N4—O2	2.4 (4)	C15—C14—Co1—N1	155.0 (2)
C2—C1—N4—Co1	0.2 (3)	C15—C14—Co1—N2	−123.5 (2)
C3—C1—N4—Co1	−177.4 (2)	C15—C14—Co1—N3	56.2 (2)
C10—C9—N5—C13	1.1 (4)	C15—C14—Co1—N5	160 (3)
C10—C9—N5—Co1	−178.9 (2)		

### Hydrogen-bond geometry (Å, °)

**Table:**

*D*—H···*A*	*D*—H	H···*A*	*D*···*A*	*D*—H···*A*
O2—H2···O4	0.84	1.67	2.479 (3)	161.
O3—H3···O1	0.84	1.67	2.478 (3)	160.
